# Association between Diet-Quality Scores, Adiposity, Total Cholesterol and Markers of Nutritional Status in European Adults: Findings from the Food4Me Study

**DOI:** 10.3390/nu10010049

**Published:** 2018-01-06

**Authors:** Rosalind Fallaize, Katherine M. Livingstone, Carlos Celis-Morales, Anna L. Macready, Rodrigo San-Cristobal, Santiago Navas-Carretero, Cyril F. M. Marsaux, Clare B. O’Donovan, Silvia Kolossa, George Moschonis, Marianne C. Walsh, Eileen R. Gibney, Lorraine Brennan, Jildau Bouwman, Yannis Manios, Miroslaw Jarosz, J. Alfredo Martinez, Hannelore Daniel, Wim H. M. Saris, Thomas E. Gundersen, Christian A. Drevon, Michael J. Gibney, John C. Mathers, Julie A. Lovegrove

**Affiliations:** 1 Hugh Sinclair Unit of Human Nutrition and Institute for Cardiovascular and Metabolic Research, University of Reading, Reading RG6 6AP, UK; r.fallaize@reading.ac.uk (R.F.); a.l.macready@reading.ac.uk (A.L.M.); 2 School of Life and Medical Sciences, University of Hertfordshire, Hatfield AL10 9AB, UK; 3 Human Nutrition Research Centre, Institute of Cellular Medicine, Newcastle University, Newcastle Upon Tyne NE1 7RU, UK; k.livingstone@deakin.edu.au (K.M.L.); carlos.celis@glasgow.ac.uk (C.C.-M.); john.mathers@newcastle.ac.uk (J.C.M.); 4 Department of Nutrition, Food Science and Physiology, University of Navarra, 31008 Pamplona, Spain; rsan.1@alumni.unav.es (R.S.-C.); snavas@unav.es (S.N.-C.); jalfmtz@unav.es (J.A.M.); 5 CIBERObn, Fisiopatología de la Obesidad y Nutrición, Instituto de Salud Carlos III, 28023 Madrid, Spain; 6 Department of Human Biology, NUTRIM School of Nutrition and Translational Research in Metabolism, Maastricht University Medical Centre +, 6200MD Maastricht, The Netherlands; cyril.marsaux@gmail.com (C.F.M.M.); w.saris@maastrichtuniversity.nl (W.H.M.S.); 7 UCD Institute of Food and Health, University College Dublin, Belfield, Dublin 4, Ireland; clare.odonovan@ucd.ie (C.B.O.); mwalsh@ndc.ie (M.C.W.); eileen.gibney@ucd.ie (E.R.G.); lorraine.brennan@ucd.ie (L.B.); mike.gibney@ucd.ie (M.J.G.); 8 ZIEL Research Center of Nutrition and Food Sciences, Biochemistry Unit, Technische Universität München, 85354 Munich, Germany; silvia.kolossa@tum.de (S.K.); hannelore.daniel@tum.de (H.D.); 9 Department of Nutrition and Dietetics, Harokopio University, 17671 Athens, Greece; gmoschi@hua.gr (G.M.); manios@hua.gr (Y.M.); 10 Microbiology and Systems Biology Group, TNO, 3704HE Zeist, The Netherlands; jildau.bouwman@tno.nl; 11 Institute of Food and Nutrition (IZZ), 02-903 Warsaw, Poland; jarosz@izz.waw.pl; 12 Instituto Madrileño de Estudios Avanzados (IMDEA) Alimentacion, 28049 Madrid, Spain; 13 Vitas Analytical Services, 0349 Oslo, Norway; teg@vitas.no; 14 Department of Nutrition, Institute of Basic Medical Sciences, Faculty of Medicine, University of Oslo, 0317 Oslo, Norway; c.a.drevon@medisin.uio.no

**Keywords:** diet scores, metabolic health, personalized nutrition, Healthy Eating Index, Mediterranean Diet Score, Dutch Healthy Diet Index, nutritional biomarkers, dried blood spots

## Abstract

Diet-quality scores (DQS), which are developed across the globe, are used to define adherence to specific eating patterns and have been associated with risk of coronary heart disease and type-II diabetes. We explored the association between five diet-quality scores (Healthy Eating Index, HEI; Alternate Healthy Eating Index, AHEI; MedDietScore, MDS; PREDIMED Mediterranean Diet Score, P-MDS; Dutch Healthy Diet-Index, DHDI) and markers of metabolic health (anthropometry, objective physical activity levels (PAL), and dried blood spot total cholesterol (TC), total carotenoids, and omega-3 index) in the Food4Me cohort, using regression analysis. Dietary intake was assessed using a validated Food Frequency Questionnaire. Participants (*n* = 1480) were adults recruited from seven European Union (EU) countries. Overall, women had higher HEI and AHEI than men (*p* < 0.05), and scores varied significantly between countries. For all DQS, higher scores were associated with lower body mass index, lower waist-to-height ratio and waist circumference, and higher total carotenoids and omega-3-index (*p* trends < 0.05). Higher HEI, AHEI, DHDI, and P-MDS scores were associated with increased daily PAL, moderate and vigorous activity, and reduced sedentary behaviour (*p* trend < 0.05). We observed no association between DQS and TC. To conclude, higher DQS, which reflect better dietary patterns, were associated with markers of better nutritional status and metabolic health.

## 1. Introduction

Healthy diets, which are characterised by high intakes of fruits, vegetables, whole grains, legumes, poultry, and seafood omega-3 fats and low intakes of refined grains and red and processed meats, are associated with a lower risk of cardiovascular diseases (CVD), such as coronary heart disease [1,2]. Although dietary recommendations aimed at preventing chronic disease mostly relate to individual nutrients or foods, it is increasingly recognised that whole dietary patterns are a more important predictor of non-communicable disease risk, including mortality from all causes and CVD [3,4].

Recently, indices have been developed to assess the overall quality (healthfulness) of dietary intakes. Typically, these indices assess intakes of 10 to 14 nutrient/dietary components, including foods such as fruits, vegetables, and oily fish, where higher intakes are healthier and foods or nutrients, for which lower intakes are healthier (e.g., processed meats, trans fatty acids and sodium). In the United States (US), the Healthy Eating Index-2010 (HEI) has been developed in accordance with the 2010 Dietary Guidelines for Americans and the US Food Guide Pyramid [5]. Higher scores of the HEI have been inversely associated with body mass index (BMI), overweight and obesity [6], and blood pressure in men [7]. Moreover, a systematic review and meta-analysis of associations between the HEI, alternate healthy eating index (AHEI) (based on food and nutrients predictive of chronic disease [8]), and Dietary Approaches to Stop Hypertension (DASH) score and disease mortality in individuals with the highest versus lowest quintile of diet quality score (DQS), revealed a significant 22% lower risk for all-cause mortality CVD and type-2 diabetes, and a 15% lower risk for cancer [9]. Independently, the AHEI has also been found to be a strong predictor of major chronic disease and CVD risk [10].

Adherence to Mediterranean diet and a healthy lifestyle has also been associated with >50% lower rate of all-cause mortality [11], and several diet-quality scores that are based on the Mediterranean diet have been developed. A 10-unit increase in the MedDietScore (MDS) (maximum score = 55) has been associated with a 4% lower 10-year risk of CHD (*p* < 0.001) [12]. Higher scores for the 14-item PREDIMED Mediterranean Diet Score (P-MDS) are significantly, and inversely, associated with adiposity indexes: BMI, waist circumference (WC), and waist-to-height ratio (WHtR) [13].

To date, DQS predominantly mimic US dietary guidance or a Mediterranean diet pattern. More recently the Dutch Healthy Diet Index (DHDI) was developed [14], which is based on the Dutch Guidelines for Healthy Diet 2006, which has been positively associated with long-chain omega-3 fatty acids (*p* trend across tertiles = 0.019) [15]. The current study used baseline data from the Food4Me study, which was a six-month web-based randomised controlled trial (RCT) designed to assess the impact of personalised dietary advice on adherence to healthy eating. The trial was conducted in a pan-European population, including seven countries (United Kingdom, Ireland, The Netherlands, Germany, Poland, Spain, and Greece) [16].

This study aims to (1) explore the association between five DQS (HEI, AHEI, MDS, P-MDS, and DHDI) and markers of cardiometabolic health (physical activity (PA), anthropometry, and dietary and metabolic markers); and, (2) compare DQS between seven Food4Me research centres in Europe.

## 2. Materials and Methods

### 2.1. Participants

A total of 1480 adults, aged 18–75 years, with complete dietary intake data at baseline, were included in the DQS analysis. Participants were recruited from the Food4Me study (www.food4me.org) between 2012 and 2014 [16]. In total, 5562 participants registered for the study (*n* = 2780 did not meet the inclusion criteria) and 1607 were randomised into the trial (*n* = 1175 excluded due to sample size being met); a further 127 dropped out prior to completing the first measurements. Exclusion criteria included absence of consent, a self-reported medically prescribed diet in the past three months, or presence of a condition likely to alter dietary requirements e.g., Crohn’s disease, coeliac disease, type-II diabetes, food allergy/intolerance, pregnancy, or lactation.

### 2.2. Study Design

The six-month Food4Me RCT was conducted by research centres in seven European Union (EU) countries: University College Dublin, Ireland, University of Reading, UK, Maastricht University, Netherlands, University of Navarra, Spain, Harokopio University, Greece, National Food and Nutrition Institute, Poland, and Technische Universität München, Germany. The RCT was designed to assess the impact of varying levels of personalised dietary advice on behaviour change and emulated a web-delivered personalised nutrition (PN) service. Participants were randomised to receive (i) standard dietary guidelines; (ii) PN based on individual diet and PA; (iii) PN based on diet, metabolic phenotype and PA; or, iv) PN based on diet, metabolic phenotype, genotype, and PA. The study was conducted online to emulate a real-life PN scenario. Further study details are published elsewhere [16,17]. The study was registered at clinicaltrials.gov (ref. NCT01530139) and was developed following international regulations and the Helsinki Declaration. Ethical approval for the study was granted at each centre and digital informed consent was obtained prior to participation.

In the present analysis, we use baseline data only. At baseline (m0), study participants received a welcome pack via post containing a dried blood spot (DBS) collection kit (Vitas Ltd, Oslo, Norway), an Isohelix SK-1 DNA buccal swab kit (LCG Genomics, Hertfordshire, UK), a TracmorD tri-axial accelerometer (Philips Consumer Lifestyle, Amsterdam, The Netherlands; http://www.directlife.philips.com), a measuring tape, and standardized instructions for completion of measurements. On the allocated study day, participants completed the validated Food4Me food frequency questionnaire (FFQ) [18,19] and Baecke PA questionnaire [20,21,22]. Participants were also instructed to collect DBS (following an 8-h fast) and buccal samples, and to measure their height (m), weight (kg) and WC (cm), according to standard procedures. Detailed instructions on how to perform the measurements were provided in printed and digital (i.e., video) format in the languages of each of the seven recruitment countries. WHtR ratio was calculated as WC/height. The TracmorD tri-axial accelerometer [23] was worn for the entire duration of the study, and data were uploaded online by participants on a bi-weekly basis. Objective physical activity, derived from the accelerometer, was recorded over a two-week period as daily physical activity level (PAL), defined as the ratio of total energy expenditure to basal metabolic rate, and time (minutes/day) spent in different intensities of PA, comprising minutes of sedentary behaviours (SB), light (LPA), moderate (MPA), and vigorous (VPA) PA; see [24] for detailed methods.

### 2.3. Dietary Assessment

The semi-quantitative validated Food4Me FFQ included questions on the consumption (frequency and portion size) of 157 food items during the past month [18,19]. Frequency of consumption was assessed by users selecting one of the following options: never or less than once a month, 1–3 times a month, once a week, 2–4 times a week, 5–6 times per week, once a day, 2–3 times per day, 4–5 times per day, and 6+ times per day. Food portion size was estimated using photographs representing small, medium, and large portions; participants could select from these images or the following written portion sizes: very small, small/medium, medium/large, or very large. Food intake (g/day) was calculated by multiplying frequency by portion size. Participants received instructions on how to fill in the FFQ prior to completing it [18].

### 2.4. Biochemical Analysis

At baseline (m0), participants collected 10 blood spots on two DBS cards (approximately 150 μL blood per card [25]). Following collection, the blood spots were dried at room temperature for 2–4 h, and placed in a sealed aluminium bag (Whatman Foil Bags, item No. 10534321, Whatman Inc., Sanford, ME, USA) containing a drying sachet (Sorb-it, item No. 10548234, Süd-Chemie, Munich, Germany). DBS cards were posted back to research centres and subsequently shipped to Vitas (Vitas Ltd., Oslo, Norway) for analysis of total carotenoids (alpha-carotene + beta-carotene + lutein + zeaxanthin + beta-cryptoxanthin + lycopene), whole blood TC using liquid chromatography-UV (LC-UV) [26] and omega-3 index ((eicosapentaenoic acid (EPA) + docosahexaenoic acid (DHA)/total fatty acids) × 100) [27]. Fatty acids were measured using gas liquid chromatography-flame ionization detection (GC-FID) [28]. A single spot was used for the analysis of each marker.

### 2.5. Diet-Quality Scores

Healthy Eating Index-2010 (HEI), Alternate Healthy Eating Index (AHEI), MedDietScore (MDS), PREDIMED Mediterranean Diet Score (P-MDS), and Dutch Healthy Diet Index (DHDI) were calculated for each participant from their FFQs.

#### 2.5.1. Healthy Eating Index-2010 (HEI)

The HEI includes 12 food groups, nine of which assess adequacy of the diet, including (1) total fruit; (2) whole fruit; (3) total vegetables; (4) greens and beans; (5) whole grains; (6) dairy; (7) total protein foods; (8) seafood and plant proteins; and, (9) fatty acids. The remaining three food groups: (10) refined grains; (11) sodium; and, (12) empty energy (i.e., energy from solid fats, alcohol, and added sugars), assess dietary components that should be consumed in moderation (reverse scoring). The maximum points allocated to each item varies (fruit and vegetable, and protein components, five points; empty energy, 20 points; remainder, 10 points) and the 12 components are summed to yield a total maximum score of 100 [5]. Sodium is scored according to classification into deciles of intake (mg/day). Application of the HEI to the Food4Me cohort has been described elsewhere [29].

#### 2.5.2. Alternate Healthy Eating Index (Ahei)

The AHEI is a 14-component scoring system comprising of seven food items: (1) fruit; (2) vegetables; (3) whole grains; (4) sugar-sweetened beverages and fruit juice; (5) nuts/legumes; (6) red/processed meat and (7) alcohol and 4 nutrients: (8) trans fatty acids; (9) polyunsaturated fatty acids; (10) long-chain omega-3 fatty acids; and, (11) sodium. Limits for whole grains (g) and alcohol (g) are differentiated according to sex. Sodium is scored according to classification into deciles of intake (mg/day). Components are weighted equally (10 points per item) and are summed to yield a maximum score of 110 [8].

#### 2.5.3. Predimed Mediterranean Diet Score (P-Mds)

The P-MDS is a 14-item questionnaire of Mediterranean Diet adherence. Items are evenly weighted (1 point per item) and comprise questions regarding use of (1) olive oil as a main culinary fat; and (2) meat preference, and adequate servings of: (3) olive oil; (4) vegetables; (5) fruit; (6) red and processed meats; (7) butter, margarine and cream; (8) sweet or carbonated beverages; (9) wine; (10) legumes; (11) fish or shellfish; (12) commercial sweets and pastries; (13) nuts; and, (14) sofrito-based dishes. The maximum score is 14 [30]. Application of the P-MDS to the Food4Me cohort has been described previously [31].

#### 2.5.4. Meddietscore (MDS)

The 11-component MDS is based on the Mediterranean diet pyramid, and assesses the frequency of consumption of key food groups. Items suggested to be consumed daily/3 times a week are scored positively (0–5 points): (1) non-refined cereals; (2) fruit; (3) vegetables; (4) legumes; (5) potatoes; (6) fish; and, (7) olive oil, whereas those that are recommended infrequently (i.e., never/once a month), were reverse scored based on consumption (5–0 points): (8) meat and meat products; (9) poultry; and, (10) full fat dairy products. Alcoholic beverage intake was scored using a non-monotonic scale, with 0 points attributed to an intake of 0 or >700 mL. The maximum score is 55 [12].

#### 2.5.5. Dutch Healthy Diet Index (Dhdi)

The DHDI is a 10-component continuous score based on 10 Dutch Guidelines for Healthy Diet 2006 [14]. Items are given a maximum score of 10 and include adequacy of: (1) physical activity; (2) vegetables; (3) fruit; (4) fish (assessed by intake of EPA and DHA) and (5) fibre; and moderate intake of (6) saturated fatty acid (SFA); (7) trans fatty acids (TFA); (8) acidic drinks and foods (ADF); (9) sodium; and, (10) alcohol (differentiated according to sex). Minimum (threshold value) and maximum scores (cut-off value) are defined for each item with those ‘recommended’ given a threshold value of 0. ADF were defined as foods and beverages that contain easily fermentable sugars and drinks that are high in food acids; this component was scored dichotomously (0, >seven occasions per day; 10 ≥ 7 occasions per day) [15].

### 2.6. Statistical Analysis

Data are shown as means ± SD. Outcome variables were checked for normal distribution and skewed data were normalised using Log_10_ (omega-3 index) and square root (TC) transformations. The differences in DQS between countries were assessed using general linear models, which were adjusted for age and sex (where significant), with post-hoc Bonferroni testing. To assess the association between DQS and markers of cardiometabolic risk, diet scores were separated into quintiles. Linear models were then used to assess trends across quintiles of diet score and BMI, WC, WHtR, DBS TC, carotenoids and omega-3 index, and PA at baseline. Associations between diet scores were evaluated using Pearson’s Rank correlation coefficient. A *p*-value of <0.05 was considered statistically significant. Data were analysed using STATA version 14 (StataCorp LP, College Station, TX, USA).

## 3. Results

Data from 1480 participants (41.6% males) were included in the present analysis, as shown in Table 1. According to BMI, participants were classified as overweight (≥25 kg/m^2^). Men (*n* = 615) had a significantly higher BMI than women (*n* = 865), as reported previously [16], and a significantly higher WC and WHtR (*p* < 0.001). Mean WC exceeded the recommended cut-off (>80 cm) in females only, indicating abdominal obesity [32]. Although PAL, SB, and LPA did not differ between sexes, men engaged in higher amounts of moderate and vigorous PA (*p* < 0.001). Blood spot TC (*p* < 0.001) was higher in men, whereas blood carotenoid concentrations were higher in women (*p* < 0.001). There were no significant differences in omega-3 index between the sexes. HEI and AHEI scores were significantly higher for women than for men (*p* < 0.001). Further socio-demographic characteristics were published previously [31].

Correlations between the DQS ranged from 0.31 to 0.63 (*p* < 0.001) (Table 2). The weakest correlation was between the Mediterranean-type diet scores, MDS and P-MDS, and strongest between the HEI and DHDI.

The association between quintiles of DQS and food and nutrient intakes are shown in Supplementary Tables S1 and S2, respectively. DQS were positively associated with intakes of fruit, vegetables, wholegrain, low fat dairy, and oily fish, and negatively associated with full-fat dairy (*p*’s trend < 0.05). With the exception of the DHDI, scores were also negatively associated with red meat intake (*p*’s trend < 0.05). Total dairy consumption was associated with higher HEI scores only (*p* < 0.001).

DQS were positively associated with intakes of PUFA, omega-3 FA, dietary fibre (DF), and folate, and negatively associated with SFA and alcohol (*p* trends < 0.05). Except for the MDS, scores were negatively associated with total fat intake and salt intake (*p* trends < 0.05). MUFA intake was positively associated with all DQS, except for the AHEI (*p* trends < 0.05). For carbohydrate, higher intakes were associated with better scores in the AHEI and DHDI (*p* trends < 0.001), and lower scores in the HEI and P-MDS (*p* trends < 0.05). The AHEI, P-MDS, and DHDI were all positively associated with total sugar intake (*p* trends < 0.001). For protein, higher intakes were positively associated with the HEI, P-MDS, and DHDI (*p* trends < 0.005), and are negatively associated with the AHEI (*p* trends = 0.008). Lastly, calcium intake was positively associated with the HEI, AHEI, and DHDI (*p* trends < 0.005), but not the Mediterranean-type DQS.

### 3.1. Comparison of Diet-Quality Scores across 7 European Countries

**Figure 1:**
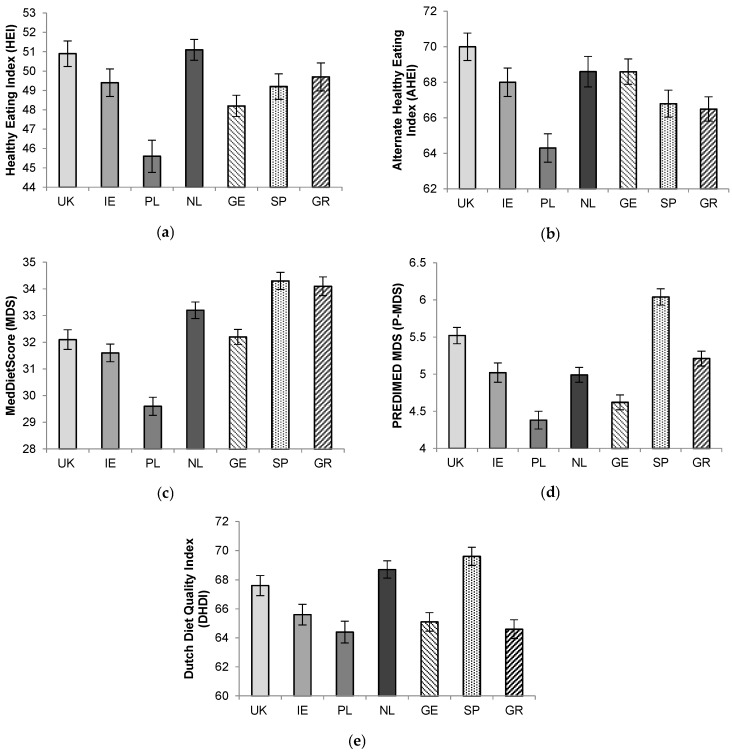
(**a**) Healthy Eating Index (HEI); (**b**) Alternate Healthy Eating Index (AHEI); (**c**) MedDietScore (MDS); (**d**) PREDIMED MDS (P-MDS); and, (**e**) Dutch Healthy Diet Index (DHDI) according to country recruiting participants to the Food4Me Study. Values represent means ± standard error of mean (SEM). UK, United Kingdom; IE, Ireland; PL, Poland; NL, The Netherlands; GE, Germany; SP, Spain; GR, Greece. Significant differences observed across all diet quality scores (*p* < 0.001) following general linear models adjusted for age and sex (HEI and AHEI): for (**a**) UK vs. PL *p* < 0.001; IE vs. PL *p* = 0.002; NL vs. PL *p* < 0.001; IE; SP vs. PL *p* < 0.001; GR vs. PL *p* < 0.001, (**b**) UK vs. PL *p* < 0.001; UK vs. SP *p* < 0.001; UK vs. GR *p* < 0.001; NL vs. PL *p* = 0.006; NL vs. SP *p* = 0.030; NL vs. GR *p* = 0.005; GE vs. PL *p* = 0.020; GE vs. GR *p* = 0.014, (**c**) SP vs. UK *p* < 0.001, SP vs. IE *p* < 0.001; SP vs. PL *p* < 0.001; SP vs. NL *p* < 0.001; SP vs. GE *p* < 0.001; GR vs. UK *p* < 0.001; GR vs. IE *p* < 0.001; GR vs. PL *p* < 0.001; GR vs. GE *p* < 0.001; NL vs. UK *p* < 0.001; IE vs. PL *p* = 0.003; GE vs. PL *p* < 0.001, (**d**) SP vs. UK *p* = 0.024; SP vs. IE *p* < 0.001; SP vs. PL *p* < 0.001; SP vs. NL *p* < 0.001, SP vs. GE *p* < 0.001; SP vs. GR *p* < 0.001; UK vs. PL *p* < 0.001; UK vs. IE *p* = 0.024; GR vs. PL *p* < 0.001; GR vs. IE *p* < 0.001; GR vs. GE *p* < 0.001; IE vs. PL *p* = 0.001; NL vs. PL *p* = 0.038, (**e**) UK vs. PL *p* < 0.001; UK vs. GE *p* = 0.002; UK vs. GR *p* < 0.001; NL vs. IE *p* = 0.022; NL vs. PL *p* < 0.001; NL vs. GE *p* < 0.001; NL vs. GR *p* < 0.001; SP vs. IE *p* < 0.001; SP vs. PL *p* < 0.001; SP vs. GE *p* < 0.001; SP vs. GR, *p* < 0.001.

Following general linear models, significant differences between countries were observed for all DQS (*p*’s < 0.001; Figure 1). Overall, there was a tendency towards higher US-type diet-quality scores (HEI and AHEI) in Northern EU countries and towards higher Mediterranean-type scores (MDS and P-MDS) in Southern EU countries. Poland had the lowest score across all the indices. HEI scores were highest in the UK and Netherlands, although these did not significantly differ from Spain, Ireland, and Greece. AHEI scores in the UK and Netherlands were significantly higher than in Greece and Spain (*p*’s < 0.05).

Spain had the highest P-MDS score, which was significantly higher than for all other centres (all *p*’s < 0.001 except for UK; *p* = 0.024). Poland had a significantly lower P-MDS than all other centres, except Germany (*p* < 0.001). For MDS, Greece and Spain both had significantly higher scores than the UK (*p*’s < 0.001), Ireland (*p*’s < 0.001), Poland (*p*’s < 0.001), and Germany (*p*’s < 0.001).

The countries with the highest DHDI were Spain, the UK, and Netherlands, which all had significantly higher DHDI scores than Germany, Poland, and Greece (*p*’s < 0.005).

### 3.2. Association between Diet-Quality Scores and Markers of Cardiometabolic Risk

The association between quintiles of DQS, physical characteristics (age, BMI, WHtR, and TC) and DBS markers are shown in Table 3 (as Q1, Q3, and Q5). Higher HEI, AHEI, MDS, and P-MDS scores, and thus diet quality, were associated with greater age (*p*’s trend < 0.001), total carotenoids (*p* trends < 0.05) and omega-3 index (*p* trends < 0.01), and lower BMI (*p* trends < 0.01), WC (*p* trends < 0.05) and WHtR (*p* trends < 0.01). Very similar BMI and WHtR were observed across quintiles of the MDS and P-MDS. For carotenoids and omega-3 index, the greatest difference between quintiles (Q5-Q1) was observed for the AHEI (0.53 µM) and P-MDS (1.00), respectively. There was no association between DQSs and TC.

Table 4 shows the association between quintiles of DQS and PA (PAL and minutes of SB, light PA, moderate PA, and vigorous PA). Higher daily PAL and vigorous PA and lower SB were associated with higher HEI, AHEI, P-MDS, and DHDI indices (*p* trend < 0.05). MDSs were not associated with PAL, yet trends for higher vigorous PA (*p* trend < 0.001) and lower SB (*p* trend = 0.004) were observed. Only the DHDI showed a significant (positive) association with minutes of light PA (*p* trend < 0.001).

## 4. Discussion

We observed a tendency for Northern EU countries to exhibit higher HEI and AHEI scores, whereas Spain displayed significantly higher Mediterranean-type DQS. All of the DQSs showed inverse associations with adiposity indexes, and positive associations with DBS total carotenoids and blood omega-3-index. Significant associations between DQS and objective PA scores were also observed.

### 4.1. Diet Quality Scores across Europe

Significantly higher MDS and P-MDS scores were observed in Spain, whereas participants in Northern-EU countries, predominantly following a Western diet, scored higher using the US-type DQS (HEI and AHEI). This is expected given that the MDS and P-MDS were developed in Southern-EU and based on dietary patterns in this region, as observed previously in the Spanish Food4Me cohort [33].

Correlations between DQS were greatest between the HEI and DHDI (*r* = 0.63). The HEI and AHEI scores also displayed a strong correlation (*r* = 0.62), similar to that reported in the 1990 Nurses’ Health study (*n* = 660; *r* = 0.60) [34], and the NIH-AARP Diet and Health Study cohort (men, *n* = 243,321, *r* = 0.62; women *n* = 182,342, *r* = 0.55) [35]. Correlations between the AHEI and HEI and Mediterranean-type scores (aMED) in the aforementioned studies ranged from 0.49–0.75, which are higher than that observed in our study (range 0.35–0.51 between MDS/P-MDS and HEI/AHEI). However, contrary to the P-MDS and MDS, which are based on epidemiological data from Southern Europe, the nine-component aMED was adapted for use in American populations [34]; thus, a stronger correlation may be expected.

### 4.2. Comparison with Previous Work

Mean HEI and AHEI scores in the 1990 Nurses’ Health study were 77 (SD 11) and 43 (SD 11), respectively [34]; the opposite pattern was observed in the Food4Me Study (HEI, 49; AHEI, 64). However, similar to findings in the present study, these DQS scores were negatively associated with BMI across quintiles of HEI and AHEI (*p*’s ≤ 0.05), and are positively associated with PA (*p*’s < 0.004) [34]. In the third NHANES cohort, the HEI was a strong independent positive predictor of all carotenoids (*p* < 0.00001) and negative predictor of BMI, plasma glucose, and HbA1c (*n* = 8719) [36], with associations also being observed between the HEI and overweight [6]. In Food4Me, none of the DQSs were associated with TC, which was also the case for the HEI in NHANES. Scores inversely associated with serum cholesterol (*p* < 0.03) in the NHANES cohort included the Recommended Food Score and Dietary Diversity Score for Recommended Foods [36], which were not explored in our present analysis. Similarly, in the SU.VI.MAX cohort (*n* = 5081), higher HEI scores were associated with healthier lifestyles and BMI in men (weak association), but not plasma lipids [7].

Low total plasma carotenoids have been reported as an independent predictor of mortality in community-dwelling adults (*n* = 1043) aged > 65 years [37]. In a case-control study of diet and breast cancer, higher HEI scores were associated with higher plasma carotenoids in women (*n* = 340) [38]. We also observed high DBS total carotenoids in the upper (5th) quartiles of HEI (1.76 µM) and AHEI (1.81 µM) in our study (>1.8 µmol/L recommended and considered ‘ideal’ in Food4Me); which is likely to reflect the ‘fruit’ and ‘vegetable’ components of the DQS. Plasma total carotenoids (excluding lycopene) have been highly correlated with total fruit and vegetable intake previously [39]. Mean DBS total carotenoids in the Food4Me Study (1.52 µmol/L), were comparable to ranges reported previously in Scandinavian cohorts (1.24–1.86 µmol/L) [40] and the European Prospective Investigation into Cancer and Nutrition (*n* = 3043 from nine EU countries) [41].

DHDI scores in the Food4Me Study (66.5 and 66.6 for men and women, respectively) were slightly higher than those that were reported in the Dutch National Food Consumption Survey (DNFCS) (*n* = 749): 57.8 for men and 60.4 for women (*p* = 0.002) [14]. Folate intake (adjusted for energy) was positively associated with the DHDI in both the DHFCS and Food4Me cohorts [14]. In the DHFCS cohort, a lack of variation in the acidic food and drink (ADF) component was observed (SD = 1.8), which led the authors to recommend adapting, or removing this component from subsequent applications of the index. In our study, the mean score for this component was 9.97 (SD 0.52) out of a maximum of 10, which supports this proposition. The DHDI was the only DQS with a positive association with calcium intake, contrary to a previous evaluation in the Dutch EFCOVAL study [15]. The EFCOVAL study also reported a lack of association between the DHDI and serum total carotenoids and TC, although the sample size (*n* = 121 adults aged 45–65 years) was smaller than in our study [15]. The DHDI is yet to be evaluated against disease outcomes.

MDS in the Food4Me cohort were greater than reported in the ATTICA cohort (*n* = 3042): 25.46 (±2.94) for men (Food4Me, 32.3; Greek participants, 33.6) and 27.18 (±3.21) for women (Food4Me, 32.5; Greek participants 34.4). This difference in scores may be explained by the high prevalence of cardiovascular risk factors in the ATTICA cohort [42]. Adherence to the MDS in the ATTICA cohort was associated with reduced prevalence of hypertension, hypercholesterolemia, diabetes, and obesity. In addition, a 10-unit increase in the diet score was associated with a 4% lower 10-year CHD risk (±0.1%, *p* < 0.001) [12]. As per the HEI and AHEI, the Mediterranean-type scores, also containing ‘fruit’ and ‘vegetable’ components, were positively associated with total carotenoids (5th quintile: MDS, 1.57 µmol/L; P-MDS, 1.77 µmol/L).

### 4.3. Implications

In our present study, all of the DQSs were positively associated with the omega-3 index (DBS), which is a reliable biomarker of n-3 status, and dietary oily fish and n-3 PUFA, EPA, and DHA intake [43,44]. This is partly expected given that the AHEI and DHDI directly assess long-chain omega-3 fatty acid intake (g/day) and EPA/DHA (mg/day), respectively, and the MDS and P-MDS assess ‘fish’ and ‘fish or shellfish’, respectively. The HEI includes components for ‘seafood and plant protein’ and ‘ratio of unsaturated to saturated fatty acids’. Mean DBS omega-3 indices in the 5th quartile of the DQSs ranged from 5.8% for MDS to 6.4% for the P-MDS; an index of 8% has been associated with the most cardio-protective effect and 4% with the greatest risk of CHD mortality [27]. The omega-3 index has been proposed as a risk factor for CHD [45]. Recently, high correlation (*r* = 0.97) has been reported between DBS, EPA, and DHA, and the omega-3 index, confirming the utility of DBS for the assessment of omega-3 index [46].

DQS were also positively associated with total PA (PAL) (except MDS) and minutes of MPA and VPA, and were negatively associated with minutes of SB. For the AHEI and P-MDS, greater diet quality (Q5) was associated with 12 min more MPA+VPA and 15 min less SB, when compared with Q1; for the DHDI, these differences were 33 and 37 min, respectively, indicating a more discriminant measure of PA. This is somewhat expected given that the DHDI is the only DQS to include PA as a scoring component [14]. Positive associations between diet quality and total PA have been reported previously for the HEI [34]. Furthermore, low PA (e.g., increased sedentary time) has been widely associated with negative health behaviours, such as lower fruit and vegetable consumption in adolescents (*n* = 11,631) [47].

In spite of lack of associations between DQS and some markers of cardiovascular risk (e.g., TC and blood pressure), the HEI and AHEI have been associated with significant risk reductions (RR) for all-cause mortality, CVD, cancer, and type-II diabetes [9]. Although no association between DBS TC and ‘diet quality’ was observed in the Food4Me Study, markers of dietary intake (e.g., total carotenoids and omega-3-index) were strongly associated with higher DQS. The observed heterogeneity of DQS across countries within the Food4Me Study suggest that certain diet scores may be more suited to particular geographical regions than others: depending on the typical dietary pattern of the population (e.g., Mediterranean or Western).

### 4.4. Strengths and Limitations

A limitation of our present study is the lack of further risk outcome measures (including LDL and HDL-C), which would enable more in-depth comparison and evaluation of the DQS. The use of DBS-derived markers for the assessment of TC differs from approaches that were used in previous studies; however, TC measured in DBS and serum display moderate to strong correlation (*r* = 0.66–0.78) [48,49]. Whilst anthropometric data were self-reported, which may have introduced error, the accuracy of Internet-based self-reported weight and height was confirmed in our previous research with strong correlations observed between self-reported and measured height (0.990 (95% CI 0.987–0.993), *p* < 0.0001) and weight (0.994 (0.991–0.995), *p* < 0.0001) [50]. In addition, sufficiently accurate self-reported WC measurements were reported in the EPIC-Oxford cohort (*n* = 4492), whereby no instructions were provided to participants [51]; in Food4Me, detailed written and digital instructions were given. Participants were recruited onto the study across the seasons (2012–2014), limiting the impact of confounding by season, although season could have influenced adherence to the diet scores (e.g., fruit more readily available in summer). Strengths of the study include a pan-European population of adults with a wide age span, a large sample size, evaluation of several DQSs from diverse geographical locations, and the use of a validated FFQ for the assessment of dietary intake [18,19]. Although, for DQS with sodium components (HEI, AHEI, DHDI), intake may have been under-estimated due to the difficulty of assessing additional sodium chloride that is added to food (e.g., during cooking, at the table) using FFQ.

## 5. Conclusions

In conclusion, DQS (HEI, AHEI, MDS, P-MDS, and DHDI) were inversely associated with markers of adiposity and sedentary activity, and positively associated with markers of dietary intake (DBS total carotenoids and omega-3 index) and total PA in the Food4Me cohort. Across the EU, Spain scored significantly higher using Mediterranean-type DQS (MDS and P-MDS) and Northern-EU countries tended to score higher with HEI and AHEI; development of an effective pan-EU DQS may be useful for directly comparing healthy eating patterns across Europe. Associations between DQS and dietary components/nutrients that were not assessed in the indices (e.g., dairy and calcium intake) may be used to determine the score most suited to a population group. Further validation of these scores against CVD risk markers in pan-EU populations might reveal valuable information.

## Figures and Tables

**Table 1:** Participant characteristics of European adults from the Food4Me Study ^a,b^.

	All	Men	Women	*p* Value
Age (years) ^c^	40 ± 13	42 ± 13	39 ± 13	<0.001
Weight (kg) ^c^	74.7 ± 15.8	83.4 ± 13.4	68.5 ± 14.4	<0.001
Height (m) ^c^	1.71 ± 0.09	1.79 ± 0.07	1.66 ± 0.07	<0.001
BMI (kg/m^2^) ^c^	25.4 ± 4.8	26.1 ± 4.1	25.0 ± 5.3	<0.001
WC (cm) ^d^	86 ± 14	93 ± 12	81 ± 13	<0.001
WHtR ^d^	0.50 ± 0.08	0.52 ± 0.07	0.49 ± 0.08	<0.001
PAL ^e^	1.73 ± 0.18	1.75 ± 0.19	1.72 ± 0.17	0.007
SB (min/day) ^e^	745 ± 77	739 ± 82	749 ± 216	0.022
Light PA (min/day) ^e^	74 ± 30	74 ± 30	74 ± 31	0.92
Moderate PA (min/day) ^e^	33 ± 20	37 ± 21	30 ± 19	<0.001
Vigorous PA (min/day) ^d^	12 ± 16	17 ± 18	8 ± 13	<0.001
Cholesterol (mmol/L) ^f^	4.6 ± 1.0	4.7 ± 1.0	4.5 ± 1.0	<0.001
Carotenoids (ug/L) ^g^	1.52 ± 0.68	1.41 ± 0.64	1.60 ± 0.70	<0.001
Omega-3 index ^h^	5.70 ± 1.21	5.72 ± 1.26	5.68 ± 1.17	0.45
HEI (0–100)	49.2 ± 9.9	47.6 ± 9.9	50.3 ± 9.7	<0.001
AHEI (0–110)	64.0 ± 10.0	62.0 ± 10.3	65.4 ± 9.6	<0.001
MDS (0–55)	32.4 ± 5.0	32.3 ± 4.9	32.5 ± 5.1	0.45
P-MDS (0–14)	5.11 ± 1.68	5.06 ± 1.76	5.16 ± 1.63	0.25
DHDI (0–100)	66.6 ± 9.2	66.5 ± 9.5	66.6 ± 9.0	0.73

^a^ BMI, body mass index; WC, waist circumference; WHtR, waist-to-height ratio; PAL, physical activity level; SB, sedentary behaviour; PA, physical activity; HEI, Healthy Eating Index; AHEI, Alternate Healthy Eating Index; MDS, MedDietScore; P-MDS, PREDIMED Mediterranean Diet Score; DHDI, Dutch Healthy Diet Index. ^b^ Values represent means ± SD. Data were analysed using general linear models. Sample sizes as follows according to total (men/women): ^c^ *n* = 1480 (615/865), ^d^ *n* = 1476 (615/861), ^e^ *n* = 1287 (539/748), ^f^ *n* = 1463 (607/856), ^g^ *n* = 1391 (584/807), ^h^ *n* = 1438 (600/838).

**Table 2:** Correlation between diet-quality scores (DQS) in European adults from the Food4Me Study ^a,b^.

	HEI	AHEI	MDS	P-MDS	DHDI
HEI	1.00				
AHEI	0.62	1.00			
MDS	0.39	0.46	1.00		
P-MDS	0.52	0.49	0.31	1.00	
DHDI	0.63	0.60	0.48	0.56	1.00

^a^ HEI, Healthy Eating Index; AHEI, Alternate Healthy Eating Index; MDS, MedDietScore; P-MDS, PREDIMED Mediterranean Diet Score. ^b^ Data are *r*-values, correlations estimated using Pearson’s Rank Correlation Coefficient, all *p*-values < 0.001.

**Table 3:** Association between quintiles of diet-quality score and markers of cardiometabolic risk in European Adults ^a,b^.

Quintiles	Age (Years)	BMI (kg/m^2^)	WHtR	WC (cm)	TC (mmol/L)	Carotenoids (µM)	Omega-3 Index
HEI							
Q1	37 ± 12	26.4 ± 5.3	0.52 ± 0.08	89 ± 15	4.5 ± 1.0	1.29 ± 0.56	5.30 ± 0.98
Q3	40 ± 13	25.5 ± 4.7	0.50 ± 0.08	86 ± 13	4.6 ± 1.0	1.55 ± 0.63	5.59 ± 1.02
Q5	42 ± 13	24.8 ± 4.2	0.49 ± 0.07	83 ± 12	4.5 ± 0.9	1.76 ± 0.77	6.27 ± 1.55
*p* for trend	<0.001	0.002	<0.001	<0.001	0.20	<0.001	<0.001
AHEI							
Q1	38 ± 13	26.97± 5.4	0.52 ± 0.08	89 ± 15	4.5 ± 1.0	1.28 ± 0.54	5.37 ± 1.05
Q3	40 ± 12	25.7 ± 4.8	0.51 ± 0.08	86 ± 13	4.6 ± 0.9	1.49 ± 0.68	5.72 ± 1.22
Q5	42 ± 14	24.0 ± 3.9	0.48 ± 0.07	82 ± 12	4.6 ± 1.0	1.81 ± 0.77	6.08 ± 1.37
*p* for trend	<0.001	<0.001	<0.001	<0.001	0.39	<0.001	<0.001
MDS							
Q1	36 ± 13	26.0 ± 5.5	0.51 ± 0.08	86 ± 15	4.4 ± 0.9	1.43 ± 0.65	5.51 ± 1.18
Q3	40 ± 13	25.0 ± 4.3	0.50 ± 0.07	85 ± 14	4.5 ± 0.9	1.56 ± 0.73	5.75 ± 1.28
Q5	43 ± 13	25.2 ± 4.4	0.50 ± 0.07	86 ± 13	4.6 ± 0.9	1.57 ± 0.67	5.84 ± 1.23
*p* for trend	<0.001	<0.001	0.002	0.013	0.70	0.017	0.001
P-MDS							
Q1	39 ± 13	25.8 ± 5.0	0.51 ± 0.08	87 ± 14	4.6 ± 1.0	1.42 ± 0.60	5.44 ± 1.04
Q3	40 ± 13	25.1 ± 4.8	0.49 ± 0.08	84 ± 14	4.5 ± 1.0	1.61 ± 0.76	5.79 ± 1.25
Q5	42 ± 13	24.9 ± 4.2	0.49 ± 0.07	84 ± 14	4.6 ± 0.9	1.77 ± 0.87	6.44 ± 1.59
*p* for trend	<0.001	0.002	<0.001	<0.001	0.16	<0.001	<0.001
DHDI							
Q1	38 ± 12	26.1 ± 5.1	0.51 ± 0.08	88 ± 14	4.5 ± 0.9	1.32 ± 0.55	5.27 ± 1.05
Q3	41 ± 13	25.6 ± 4.3	0.50 ± 0.07	86 ± 13	4.6 ± 1.0	1.54 ± 0.67	5.66 ± 1.19
Q5	42 ± 14	23.7 ± 4.5	0.47 ± 0.06	81 ± 11	4.4 ± 0.9	1.83 ± 0.79	6.23 ± 1.31
*p* for trend	<0.001	<0.001	<0.001	<0.001	0.32	<0.001	<0.001

^a^ BMI, body mass index; WC, waist circumference; WHtR, waist-to-height ratio; TC, total cholesterol; HEI, Healthy Eating Index; AHEI, Alternate Healthy Eating Index; MDS, MedDietScore; P-MDS, PREDIMED Mediterranean Diet Score; DHDI, Dutch Healthy Diet Index ^b^ Values represent mean ± SD, data analysed using linear regression across quintiles of DQS. Models adjusted for sex, age, country, energy intake (kcal), objective PAL.

**Table 4:** Association between quintiles of diet-quality score and physical activity levels (PAL) in European adults from the Food4Me study ^a,b^.

Quintiles	PAL	SB (min/day)	Light PA (min/day)	Moderate PA (min/day)	Vigorous PA (min/day)
HEI					
Q1	1.71 ± 0.17	746 ± 78	75 ± 32	32 ± 21	10 ± 15
Q3	1.73 ± 0.18	750 ± 70	74 ± 29	33 ± 21	10 ± 14
Q5	1.76 ± 0.18	727 ± 76	76 ± 33	34 ± 19	14 ± 19
*p* for trend	<0.001	0.001	0.74	<0.001	<0.001
AHEI					
Q1	1.71 ± 0.14	750 ± 76	74 ± 32	33 ±19	9 ± 12
Q3	1.72 ± 0.18	748 ± 76	73 ± 29	32 ± 23	11 ± 14
Q5	1.78 ± 0.21	735 ± 76	74 ± 30	36 ± 20	17 ± 20
*p* for trend	<0.001	0.003	0.26	0.002	<0.001
MDS					
Q1	1.72 ± 0.17	745 ± 83	74 ± 32	32 ± 20	10 ± 14
Q3	1.74 ± 0.18	743 ± 73	74 ± 32	34 ± 21	13 ± 17
Q5	1.74 ± 0.18	744 ± 74	76 ± 31	34 ± 20	12 ± 15
*p* for trend	0.10	0.022	0.53	0.019	0.011
P-MDS					
Q1	1.72 ± 0.16	745 ± 77	74 ± 30	34 ± 21	10 ± 12
Q3	1.73 ± 0.17	747 ± 77	74 ± 32	32 ± 19	11 ± 16
Q5	1.78 ± 0.87	730 ± 77	74 ± 30	36 ± 20	19 ± 20
*p* for trend	<0.001	0.004	0.71	0.10	<0.001
DHDI					
Q1	1.67 ± 0.13	757 ± 74	70 ± 29	27 ± 16	7 ± 9
Q3	1.73 ± 0.18	747 ± 78	74 ± 30	31 ± 20	10 ± 15
Q5	1.85 ± 0.22	720 ± 70	80 ± 30	44 ± 22	22 ± 23
*p* for trend	<0.001	<0.001	<0.001	<0.001	<0.001

^a^ PAL, physical activity level; SB, sedentary behaviour; PA, physical activity; HEI, Healthy Eating Index; AHEI, Alternate Healthy Eating Index; MDS, MedDietScore; P-MDS, PREDIMED Mediterranean Diet Score; DHDI, Dutch Healthy Diet Index ^b^ Values represent means ± SD, data analysed using linear regression across quintiles of DQS. Models adjusted for sex, age, country, and energy intake (kcal).

## Supplementary Materials

The following are available online at http://www.mdpi.com/2072-6643/10/1/49/s1, Table S1: Association between quintiles of diet-quality score and food intake in European Adults, Table S2: Association between quintiles of diet-quality score and nutrient intake in European Adults.
