# Supplementary material for: Association between Diet-Quality Scores, Adiposity, Total Cholesterol and Markers of Nutritional Status in European Adults: Findings from the Food4Me Study
Source: Nutrients. 2018 Jan 6;10(1):49. doi: 10.3390/nu10010049 (PMC5793277; doi:10.3390/nu10010049)
Supplement: Supplementary file 1 [file nutrients-10-00049-s001.zip › Nutrients_DQS_EU_Fallaize et al._Supplementary/Nutrients_DQS_EU_Fallaize et al._S1.docx]

**Table S1.** Association between quintiles of diet-quality score and food intakes in European Adults^a,b^

| Food (g/day) |  | HEI | AHEI | MDS | P-MDS | DDQI |
| --- | --- | --- | --- | --- | --- | --- |
| Fruit | Q1 | 283 ± 258 | 257 ± 203 | 276 ± 232 | 265 ± 238 | 190 ± 185 |
|  | Q5 | 421 ± 267 | 514 ± 315 | 470 ± 312 | 578 ± 357 | 572 ± 363 |
|  | *P* | <0.001 | <0.001 | <0.001 | <0.001 | <0.001 |
| Vegetables | Q1 | 135 ± 85 | 152 ± 88 | 171 ± 130 | 150 ± 94 | 130 ± 86 |
|  | Q5 | 324 ± 192 | 313 ± 165 | 287 ± 166 | 393 ± 247 | 311 ± 173 |
|  | *P* | <0.001 | <0.001 | <0.001 | <0.001 | <0.001 |
| Wholegrain | Q1 | 107 ± 160 | 128 ± 192 | 139 ± 197 | 160 ± 168 | 107 ± 113 |
|  | Q5 | 210 ± 151 | 213 ± 159 | 193 ± 175 | 199 ± 169 | 267 ± 212 |
|  | *P* | <0001 | <0.001 | 0.004 | 0.013 | <0.001 |
| Red meat | Q1 | 98 ± 97 | 123 ± 103 | 80 ± 66 | 91 ± 90 | 93 ± 82 |
|  | Q5 | 60 ± 53 | 40 ± 34 | 77 ± 69 | 55 ± 38 | 55 ± 50 |
|  | *P* | 0.022 | <0.001 | 0.19 | <0.001 | <0.001 |
| Dairy | Q1 | 266 ± 222 | 343 ± 321 | 322 ± 280 | 315 ± 255 | 301 ± 243 |
|  | Q5 | 353 ± 271 | 300 ± 228 | 317 ± 229 | 324 ± 290 | 353 ± 278 |
|  | *P* | <0.001 | 0.38 | 0.21 | 0.36 | 0.068 |
| Dairy (full-fat) | Q1 | 99 ± 148 | 117 ± 228 | 123 ± 232 | 102 ± 175 | 119 ± 203 |
|  | Q5 | 47 ± 72 | 53 ± 78 | 56 ± 86 | 56 ± 75 | 54 ± 83 |
|  | *P* | 0.001 | <0.001 | <0.001 | <0.001 | <0.001 |
| Dairy (low-fat) | Q1 | 168 ± 191 | 226 ± 265 | 199 ± 200 | 213 ± 223 | 182 ± 180 |
|  | Q5 | 307 ± 268 | 247 ± 215 | 261 ± 230 | 268 ± 290 | 298 ± 263 |
|  | *P* | <0.001 | 0.001 | 0.019 | 0.002 | <0.001 |
| Oily fish | Q1 | 12.1 ± 17.8 | 15.2 ± 23.7 | 12.0 ± 17.8 | 11.3 ± 14.2 | 7.1 ± 10.3 |
|  | Q5 | 32.7 ± 31.2 | 26.3 ± 25.2 | 33.6 ± 29.7 | 33.4 ± 32.5 | 39.1 ± 29.2 |
|  | *P* | <0.001 | <0.001 | <0.001 | <0.001 | <0.001 |

^a^ Data analysed using linear regression. Models adjusted for sex, age, energy intake (kcal) and country. HEI, Healthy Eating Index; AHEI, Alternate Healthy Eating Index; MDS, MedDietScore; P-MDS, PREDIMED Mediterranean Diet Score; DHDI, Dutch Healthy Diet Index ^b^ Values represent mean ± SD, data analysed using linear regression across quintiles of DQS. Models adjusted for sex, age, country, energy intake (kcal), objective PAL.
