# Supplementary material for: Association between Diet-Quality Scores, Adiposity, Total Cholesterol and Markers of Nutritional Status in European Adults: Findings from the Food4Me Study
Source: Nutrients. 2018 Jan 6;10(1):49. doi: 10.3390/nu10010049 (PMC5793277; doi:10.3390/nu10010049)
Supplement: Supplementary file 1 [file nutrients-10-00049-s001.zip › Nutrients_DQS_EU_Fallaize et al._Supplementary/Nutrients_DQS_EU_Fallaize et al._S2.docx]

**Table S2.** Association between quintiles of diet-quality score and nutrient intake in European Adults^a,b^

| Nutrient |  | HEI | AHEI | MDS | P-MDS | DDQI |
| --- | --- | --- | --- | --- | --- | --- |
| Total FA (%TE) | Q1 | 36.5 ± 6.1 | 36.7 ± 7.1 | 35.9 ± 6.5 | 37.0 ± 5.6 | 38.4 ± 5.6 |
|  | Q5 | 35.8 ± 6.2 | 35.7 ± 5.5 | 35.7 ± 5.6 | 34.5 ± 6.9 | 32.5 ± 4.8 |
|  | *P* | 0.022 | 0.006 | 0.79 | <0.001 | <0.001 |
| SFA (%TE) | Q1 | 15.8 ± 3.5 | 15.3 ± 3.4 | 15.0 ± 3.7 | 15.6 ± 3.2 | 16.3 ± 3.0 |
|  | Q5 | 12.2 ± 2.5 | 13.0 ± 2.8 | 13.0 ± 2.5 | 11.9 ± 2.8 | 11.4 ± 1.9 |
|  | *P* | <0.001 | <0.001 | <0.001 | <0.001 | <0.001 |
| PUFA (%TE) | Q1 | 5.1 ± 1.2 | 5.2 ± 1.2 | 5.5 ± 1.5 | 5.5 ± 1.4 | 5.6 ± 1.6 |
|  | Q5 | 6.5 ± 1.9 | 6.5 ± 1.7 | 5.8 ± 1.4 | 6.2 ± 1.7 | 5.9 ± 1.5 |
|  | *P* | <0.001 | <0.001 | 0.007 | <0.001 | 0.002 |
| MUFA (%TE) | Q1 | 13.3 ± 2.7 | 13.8 ± 3.0 | 13.0 ± 2.8 | 13.6 ± 2.8 | 14.3 ± 2.8 |
|  | Q5 | 14.7 ± 3.9 | 13.8 ± 3.1 | 14.5 ± 3.4 | 13.9 ± 3.9 | 12.8 ± 3.0 |
|  | *P* | <0.001 | 0.46 | <0.001 | 0.015 | <0.001 |
| Omega-3 FA (%TE) | Q1 | 0.57 ± 0.15 | 0.62 ± 0.17 | 0.67 ± 0.26 | 0.61 ± 0.16 | 0.62 ± 0.20 |
|  | Q5 | 0.93 ± 0.36 | 0.82 v 0.29 | 0.80 ± 0.25 | 0.97 ± 0.35 | 0.87 ± 0.33 |
|  | *P* | <0.001 | <0.001 | <0.001 | <0.001 | <0.001 |
| Carbohydrate (%TE) | Q1 | 47.5 ± 7.1 | 44.7 ± 8.1 | 45.7 ± 8.8 | 46.3 ± 7.2 | 43.1 ± 7.1 |
|  | Q5 | 44.3 ± 7.7 | 47.3 ± 6.7 | 46.6 ± 6.7 | 45.2 ± 8.3 | 49.8 ± 6.6 |
|  | *P* | <0.001 | <0.001 | 0.059 | 0.030 | <0.001 |
| Sugar (%TE) | Q1 | 21.2 ± 6.0 | 19.4 ± 5.9 | 21.1 ± 6.1 | 20.2 ± 5.8 | 18.9 ± 5.7 |
|  | Q5 | 21.6 ± 5.7 | 23.5 ± 5.5 | 21.2 ± 5.3 | 22.8 ± 5.7 | 23.5 ± 6.1 |
|  | *P* | 0.53 | <0.001 | 0.96 | <0.001 | <0.001 |
| Dietary fibre (g) | Q1 | 25.8 ± 13.5 | 26.1 ± 15.3 | 24.3 ± 14.4 | 25.6 ± 12.3 | 20.9 ± 9.2 |
|  | Q5 | 32.0 ± 14.0 | 35.2 ± 13.8 | 34.9 ± 13.6 | 39.3 ± 16.9 | 39.7 ± 17.0 |
|  | *P* | <0.001 | <0.001 | <0.001 | <0.001 | 0.018 |
| Protein (%TE) | Q1 | 15.2 ± 2.8 | 17.3 ± 3.9 | 17.3 ± 4.2 | 16.3 ± 3.4 | 16.9 ± 3.8 |
|  | Q5 | 19.2 ± 4.6 | 16.3 ± 3.1 | 17.2 ± 2.5 | 18.4 ± 4.5 | 17.7 ± 3.7 |
|  | *P* | <0.001 | 0.008 | 0.80 | <0.001 | 0.026 |
| Alcohol (%TE) | Q1 | 3.6 ± 4.9 | 3.6 ± 4.8 | 3.5 ± 5.0 | 2.7 ± 3.2 | 4.0 ± 4.4 |
|  | Q5 | 2.6 ± 2.5 | 2.9 ± 2.2 | 2.7 ± 2.6 | 4.3 ± 4.7 | 2.2 ± 2.0 |
|  | *P* | 0.019 | 0.004 | <0.001 | <0.001 | <0.001 |
| Salt (g) | Q1 | 8.63 ± 4.80 | 9.02 ± 4.75 | 7.18 ± 4.02 | 7.62 ± 3.89 | 7.29 ± 4.0 |
|  | Q5 | 5.75 ± 2.47 | 6.14 v 2.41 | 7.58 ± 3.37 | 7.05 ± 2.77 | 7.27 ± 3.7 |
|  | *P* | <0.001 | <0.001 | 0.28 | <0.001 | <0.001 |
| Calcium (mg) | Q1 | 1337 ± 705 | 1412 ± 749 | 1239 ± 650 | 1254 ± 604 | 1201 ± 553 |
|  | Q5 | 1148 ± 543 | 1203 ± 483 | 1316 ± 560 | 1329 ± 581 | 1345 ± 658 |
|  | *P* | <0.001 | 0.020 | 0.28 | 0.14 | 0.004 |
| Folate (μg) | Q1 | 388 ± 208 | 409 ± 228 | 380 ± 207 | 377 ± 188 | 346 ± 188 |
|  | Q5 | 422 ± 198 | 437 ± 185 | 462 ± 209 | 493 ± 323 | 503 ± 228 |
|  | *P* | <0.001 | <0.001 | <0.001 | <0.001 | 0.004 |

^a^ Data analysed using linear regression. Models adjusted for sex, age, energy intake (kcal) and country. HEI, Healthy Eating Index; AHEI, Alternate Healthy Eating Index; MDS, MedDietScore; P-MDS, PREDIMED Mediterranean Diet Score; DHDI, Dutch Healthy Diet Index; FA, fatty acid; SFA, saturated fatty acids; MUFA, monounsaturated fatty acids; PUFA, polyunsaturated fatty acids ^b^ Values represent mean ± SD, data analysed using linear regression across quintiles of DQS. Models adjusted for sex, age, country, energy intake (kcal), objective PAL.
